# Cyberloafing to Escape From the “Devil”: Investigating the Impact of Abusive Supervision From the Third-Party Perspective

**DOI:** 10.3389/fpsyg.2021.722063

**Published:** 2022-02-04

**Authors:** Xuedong Liang, Gengxuan Guo, Qunxi Gong, Sipan Li, Ziyang Li

**Affiliations:** ^1^School of Business, Sichuan University, Chengdu, China; ^2^The Economy and Enterprise Development Institute, Sichuan University, Chengdu, China

**Keywords:** peer abusive supervision, negative affectivity, cyberloafing, hostile attribution bias, affective events theory

## Abstract

**Purpose:**

Previous studies on cyberloafing focus on individual and organization factors, ignoring the situation of employes as the event observers. Drawing on affective events theory (AET), the present study proposed a theoretical model for the relationships among peer abusive supervision, negative affectivity, cyberloafing, and hostile attribute bias, which aims to bridge the above research gap.

**Methodology:**

Multiwave data of 355 employes from 8 service-oriented companies in Southwest China supported our model. Time-lag method and critical incident techniques were introduced during the data collection stage. Ordinary least squares (OLS) regression and bootstrapping method were employed for hypothesis test.

**Findings:**

The empirical results indicated that peer abusive supervision was positively related to third party’s cyberloafing, and the third party’s negative affectivity plays a mediating role among the above relationships. In addition, the third party’s hostile attribution bias moderated the mediating role of third party’s negative affectivity. Specifically, the effect of peer abusive supervision on third party’s negative affectivity and the mediating effect of this negative affectivity were stronger when the third party’s hostile attribution bias was higher.

**Originality:**

Drawing on AET, the current study constructed a process model of third party’s cyberloafing reactions to peer abusive supervision, which helps explain the affective mechanism and the boundary conditions of the above “events-affectivity-behavior” path. Our model is a positive response to previous scholars’ calls for research of abusive supervision from multiple perspectives. Meanwhile, the current study explored the antecedent variable of cyberloafing from the perspective of event observers, which provides a theoretical basis for follow-up-related research. Thirdly, this study further expanded the theoretical boundaries of AET.

## Introduction

Cyberloafing refers to the counterproductive work behavior (CWB) in which employes check private emails and browse non-work-related websites during working hours, thereby affecting their work progress (Lim, 2002; Askew et al., 2014). During these years, with the continuous development of information technology, cyberloafing has widely existed on a global workplace and brought a negative impact to enterprises (Baturay and Toker, 2015; Usman et al., 2021). A recent survey showed that 89% of employes waste time at work every day in ways of visiting various web pages for personal purposes (Salary.com, 2014). In the United States, the annual loss caused by employes engaged in cyberloafing is as high as 85 billion United States dollars (Wagner et al., 2012). In particular, with the protection policies such as social distancing and lockdown due to the coronavirus disease (COVID-19), working from home has become more and more popular (Shao et al., 2021; Zhang et al., 2021). Using computers to work remotely at home allows employes to have more work autonomy, which makes them possible to engage in more cyberloafing (O’Neill et al., 2014a). Considering the destructive effects of cyberloafing, especially during the current pandemic, companies need to take measures to avoid cyberloafing by employes in the organization. Forasmuch, it is necessary to clarify the driving factors of cyberloafing, and then formulate regulations and training plans to reduce the frequency of cyberloafing within the organization.

Motivated by reducing the potential costs caused by cyberloafing, previous literatures have conducted rich investigation on the driving factors of cyberloafing, which can be roughly summarized into the following two aspects. (1) Individual factors: Sociodemographics (e.g., gender, age, and tenure) (Cheng et al., 2020; Hensel and Kacprzak, 2020), personality traits (e.g., big five, locus of control, emotional stability, and honesty) (O’Neill et al., 2014b; Yildiz Durak and Saritepeci, 2019), emotion (e.g., empathetic concern and anger) (Zoghbi Manrique de Lara, 2006; Zhang et al., 2019), and habits (e.g., past experience, ethical judgment, and tendency etc.) (Khansa et al., 2017; Batabyal and Bhal, 2020) have been regarded as the key individual factors affecting employe cyberloafing. (2) Organizational factors: Organizational infrastructure (Askew and Buckner, 2017), organizational culture (e.g., hierarchy, justice, and meaningful work) (Zoghbi-Manrique-de-Lara and Viera-Armas, 2017; Khansa et al., 2018; Usman et al., 2021), and monitoring strategies (Hensel and Kacprzak, 2021) are key antecedent variables of employe cyberloafing. Scholars have made a rich and useful exploration of the antecedent variables of cyberloafing. However, the extant studies on the antecedent of cyberloafing still exists some room for further development. As one of the most important situational factors shaping employes’ workplace behavior, supervisors play a key role in promoting employes’ career development and improving organizational performance (Ouyang et al., 2015; Mei et al., 2021). However, prior literatures in the organizational context did not fully integrate the supervisory factor into the research of employe cyberloafing (Kim et al., 2016). Since the supervisor is located in the power center of the team, their behaviors have an important influence on employes, especially their negative behaviors, which are likely to bring unexpected consequences and even bring a series of ripple effects (Zhang and Liu, 2018; Dhanani and Lapalme, 2019). Therefore, it is necessary to investigate the supervisor’s behavioral impact on employes’ cyberloafing.

At present, some emerging literatures have launched preliminary exploration on this topic. For example, Agarwal and Avey (2020) suggested that abusive supervision will reduce employes’ psychological capital and induce their cyberloafing, and the psychological contract breach they perceived will reinforce this negative impact. Lim et al. (2021) hold that abusive supervision will drive employes to implement cyberloafing by increasing their emotional exhaustion, and organizational commitment will alleviate this negative effect. Abusive supervision refers to the hostile verbal and non-verbal behaviors performed by supervisors against subordinates, such as mocking and ridicule, openly scolding, and deliberately neglecting, in addition to physical contact behaviors (Tepper, 2000). Compared with supervisors’ other negative behaviors, abusive supervision does not include physical contact, which means that it is more common in contemporary workplaces and has universal research value (Einarsen et al., 2007; Fischer et al., 2021). However, when we analyze abusive supervision, we will find that there are three parties in the incident: the supervisor (behavior perpetrator), the abused employe (behavior receiver), and the third party (behavior observer). Therefore, the above literatures only examined the impact of supervisors’ behavior on victim employes’ cyberloafing, ignoring its influence on the event observers. The degree to which a coworker is being abused by his/her supervisor perceived by the third party can be defined as peer abusive supervision (Peng et al., 2013). Although the antecedent variable of cyberloafing from the perspective of event observer is an indirect effect, it is often a majority effect, which means that employes play the role of observers rather than event participants for most times of their work (Landers and Callan, 2014). The latest review on cyberloafing also urges future research to examine the antecedent variables of cyberloafing from the perspective of coworker, which is the third party in the present article (Tandon et al., 2021). Therefore, it is necessary and urgent to examine the impact of peer abusive supervision on third parties, who are the largest group in the organization. In summary, the current study aims to investigate the following theoretical issues: *Whether (main effect), how (mediating mechanism), and when (boundary condition) will peer abusive supervision trigger third party’s cyberloafing?*

**FIGURE 1:**
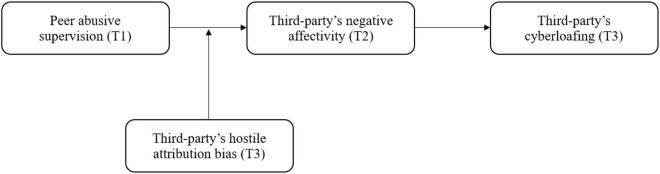
Theoretical framework of current study.

In order to advance the study on the driving factors of cyberloafing from a third-party perspective, the present article adopts affective events theory (AET) to investigate the impacts of peer abusive supervision on observer’s cyberloafing. The AET holds that work events are the main source of individuals affectivities, which will trigger their affective reactions, and the resulting affective state will further influence an individual’s subsequent attitudes and behaviors (Weiss and Cropanzano, 1996). Hence, after witnessing peer abusive supervision, the third party’s cyberloafing reactions will not be achieved overnight. According to AET’s cognitive judgment approach, when the negative affective event of peer abusive supervision is triggered, the third-party employes will first conduct a cognitive evaluation, that is to attribute the supervisor’s motives for implementing abusive supervision, and then go through a process of “cognitive evaluation- affective response- behavior reaction” (Weiss and Cropanzano, 1996). Past literatures have shown that when individuals experience negative affective events, they often also produce negative affectivity (Rodell and Judge, 2009; Wang and Li, 2019). Peer abusive supervision, as the third party’s negative affective work events, will pose threats and challenges to third-party employes that will result in their negative affectivity, which may make them get away from the supervisors for fleeing the negative affectivity. Cyberloafing is an activity that can help employes get rid of stress and negative affectivity (Jo Ann, 2019). Some similar studies also provide indirect evidence of individual negative affectivity as the mediating variable (Seo et al., 2004; Rodell and Judge, 2009). Therefore, the third party’s negative affectivity helps to answer “how” peer abusive supervision can cause third party’s cyberloafing. Further, the AET suggests personal traits related to affective events at work will influence an individual’s affective response and his/her subsequent behavior reaction (Weiss and Cropanzano, 1996). Specific to the theoretical model of this study, as an unethical behavior, whether and how peer abusive supervision affects third party’s negative affectivity depends on his/her attribution to the supervisor’s behavioral motivation and his/her sensitivity to the supervisor’s negative behavior, that is, how the third party assesses the supervisor’s motives for abusing his/her colleagues (Yu and Duffy, 2021). The hostile attribution bias, which refers that individuals prefer attributing errors, responsibilities, and injuries to others so as to blame them for their negative behavior, satisfies this personality trait (De Castro et al., 2002). Individuals with high-level hostile attribution bias tend to interpret others unfriendly behavior as their hostile motives, even if this is not the case (Matthews and Norris, 2002). After witnessing peer abusive supervision, the third parties with the above characteristics tend to produce more negative affectivity and engage in more cyberloafing. Therefore, the third party’s hostile attribution bias helps to answer “when” peer abusive supervision can cause third party’s negative affectivity and cyberloafing. In conclusion, by constructing the above moderated mediation model, the present study explores the internal mechanism (negative affectivity) and boundary conditions (hostile attribution bias) of peer abusive supervision on third party’s cyberloafing. Our theoretical model is presented in Figure 1.

The current article makes the following theoretical contributions. First, by shifting the focus to observer, the current study emphasizes that peer abusive supervision, a negative affective event, can bring threat perceptions and induce negative affectivity to third party, who will further stay away from this kind of negative state through cyberloafing. Our work reveals the impact of abusive supervision on another important group in the workplace, which is not only a useful supplement to the research on abusive supervision from a third-party perspective, but also a positive response to previous scholars’ calls for research from multiple perspectives (Harris et al., 2013; Tepper et al., 2017). Second, the present study found new antecedent variables that affect employe cyberloafing. Previous studies on cyberloafing focused on the two aspects of employe personality traits and interpersonal interaction from the perspective of participants ignoring the role of employes as events bystanders (Cheng et al., 2020). Our research shows that when the third party witness his/her colleagues being abused, he/she will have negative affectivity and will choose cyberloafing to stay away from this negative state. To our knowledge, the current study is the first article to explore the antecedent variable of cyberloafing from the perspective of event observers, which provides a theoretical basis for follow-up-related research. Third, this study further expanded its theoretical boundaries based on the AET. Based on the AET, we found that the third party’s hostile attribution bias will moderate the effect of peer abusive supervision on his/her negative affectivity. Specially, for employes with high-level hostile attribution bias, the negative impact of peer abusive supervision on third party is more serious. Therefore, hostile attribution bias moderates the impact of the affective events on third parties. From this perspective, the hostile attribution bias of the third party may be a theoretical boundary of affective events influencing his/her own cognition and affectivity.

## Theory and Hypotheses

### Affective Events Theory

The affective events theory suggests that an event happened at a specific time and a specific place can be regarded as the affective event, especially these important events (Weiss and Cropanzano, 1996). As one of the core constructs in AET, affective events have the following three typical characteristics: (1) The event occurred in an organization; (2) The event can trigger an individual’s affective response; and (3) The event should be related to personal goals or work. Work events are the main source of individual affectivity, which will trigger the affective reactions of employes, and the resulting affective state will further influence the individual’s subsequent attitudes and behaviors (Weiss et al., 1999). The AET has been widely used to explain the employes’ affective reactions in workplace (Judge et al., 2006), especially in explaining the influence of the interpersonal interaction in the workplace on individual affectivity. For example, researchers have found that positive work events and good interpersonal relationships can make employes have positive affectivity, while negative work events and interpersonal conflicts are the main reasons for individuals to produce negative affectivity (Dimotakis et al., 2011; Bono et al., 2012).

Drawing on AET, combined with relevant research on peer abusive supervision (Mitchell et al., 2015), abusive supervision behavior, which is an obviously harmful behavior to the interests of the organization and other members, is an important type of affective events ubiquitous in the workplace, so it naturally constitutes the affective event of the observer employe. When a third party observes that his colleague is being abused, his own cognitive assessment of the work event (i.e., peer abusive supervision) will also influence his affective response, which will further affect the corresponding work outcomes. Therefore, the AET provides a suitable theoretical explanation framework for exploring the impact of abusive supervision event on third-party employes.

### Peer Abusive Supervision and Third Parties’ Cyberloafing

Drawing on AET, we suggest that peer abusive supervision will trigger third party’s cyberloafing. The AET holding that individuals will perform cognitive evaluation under the stimulation of affective events, and related evaluation results will trigger a series of behavioral responses (Weiss and Cropanzano, 1996). Abusive supervision is manifested in mocking, ridiculing, and deliberately neglecting the victims, which to a large extent violate the ethics of the workplace (Tepper, 2000). Through the above definition, we find that peer abusive supervision happens in the organization and is closely related to the work of the observer, which can constitute an affective event of the third party. Therefore, as an important affective event for third party, peer abusive supervision may affect the observers’ behavior tendency. Specially, when the third-party employe perceives the peer abusive supervision, he may express sympathy to the abused colleague, question the workplace’s professional norms, and doubt his own future treatment at the same time (O’Reilly and Aquino, 2011; Chen et al., 2020). In response to the above organizational injustices, the third party may be unwilling to show proactive behavior for the benefit of the organization. On the contrary, they may adopt more passive attitudes and behaviors, which may result themself being indifferent to the problems of the organization, and even showing schadenfreude (Shao et al., 2018; Xu et al., 2020). Therefore, cyberloafing may be the direct choice for observers in the face of peer abusive supervision events for this behavior’s relative safety (Zhang et al., 2019).

Cyberloafing refers to the voluntary behavior of employes using the Internet for non-work purposes during working hours, which has been considered as a kind of counterwork behavior for its undermining performance (Lim, 2002; Koay Kian, 2018). Cyberloafing is difficult to detect by supervisors because it does not require employes to leave their desks (Wagner et al., 2012). In addition, cyberloafing will give the supervisor the illusion that the employe is working hard for his being concentrating on the computer screen. In fact, the third party is just using the company network to handle private affairs. Therefore, when the third party perceive peer abusive supervision, they may choose cyberloafing, a relatively safe behavior, to retaliate against their supervisors.

In summary, we propose the following hypothesis:

*Hypothesis 1: Peer abusive supervision has the positive effect on third party’s cyberloafing.*

### Negative Affectivity as the Mediating Mechanism

According to the AET, affective events can induce an individual’s affective response, which is a proximal outcome variable of the affective events, that will affect an individual’s behavior, as well (Weiss and Cropanzano, 1996). The AET provides an over-arching theoretical framework for explaining the antecedents and results of an individual’s affective reactions in workplace, which may further induce individual behaviors (Ashkanasy et al., 2014). Therefore, the negative affectivity of a third party is likely to play a mediating role in the influence of peer abusive supervision (affective events) on cyberloafing.

#### Peer Abusive Supervision and Third Party’s Negative Affectivity

To explore the third party’s affective response to peer abusive supervision, we regard the third party’s negative affectivity as their emotional state, which refer to an individual’s affective feelings at a given time (Thoresen et al., 2003; Wong et al., 2006). Negative affectivity describes the instantaneous affective response of an individual to a specific experience at a specific time, such as anger, tension, or fear (Seo et al., 2004). When individuals encounter setbacks or negative life events, they often produce negative affectivity (Grandey et al., 2002). The current study suggests that negative affectivity is likely to be the negative response to the peer abusive supervision. According to the AET, affective events may induce affective reactions through the individual’s cognitive evaluation mechanism (Weiss and Cropanzano, 1996). In particular, the direction of affective response is closely related to the characteristics of affective events, and individuals will make positive or negative judgments based on the consistency between the event and their own goals (Dasborough, 2006). When affective events cannot meet personal needs or values, negative affectivity will appear (Glasø et al., 2011).

In the workplace, justice and civilized workplace ethics are one of the basic goals pursued by employes (Colquitt et al., 2001). As a negative affective event, peer abusive supervision poses challenges and threats to the resources of third party for it is violating the deontic justice principle (Priesemuth and Schminke, 2017). Therefore, witnessing abusive supervision is likely to trigger the third party’s lower perceptions of workplace collegiality, which may further create uncertainty about the treatment of others and of oneself (Reich and Hershcovis, 2015). Therefore, we suggest that peer abusive supervision cannot meet the workplace requirements of third party, which may further arouse his/her negative affectivity.

In summary, we propose the following hypothesis:

*Hypothesis 2: Peer abusive supervision has a positive effect on third party’s negative affectivity.*

#### Third Party’s Negative Affectivity and Cyberloafing

At the same time, negative affectivity will further affect the third party’s cyberloafing. Drawing on the AET, affective events can arouse the affectivity of third-party employes and further drive their behavior (Weiss and Cropanzano, 1996). From this perspective, the follow-up behavior of third-party employes is likely to be a negative response to their negative affectivity. Second, the negative affectivity that accompany the bad experience has a priming extension effect on the individual’s cognition, and third-party employes may respond with negative behaviors (Mor and Winquist, 2002). Specially, in order to avoid the further deepening of negative affectivity, the third-party employes may be motivated to cyberloafing, through which the third party may gain positive affectivity *via* social interaction with others on the internet, to avoid direct contact with the abusive supervisor (Kim et al., 2016).

As mentioned earlier, we suggest that peer abusive supervision will pose threats and challenges to third-party employes that will result in their negative affectivity, which may make them get away from the supervisors for fleeing the negative affectivity. According to Jo Ann (2019), cyberloafing is an activity that can help employes get rid of stress and negative affectivity. Therefore, we suggest that the third-party employes who have negative affectivity due to peer abusive supervision are more likely to go cyberloafing to cope with the above harassment.

In summary, we propose the following hypothesis:

*Hypothesis 3: Third party’s negative affectivity has the positive effect on third party’s cyberloafing.*

*Hypothesis 4: Third party’s negative affectivity plays a mediating role between peer abusive supervision and cyberloafing.*

### Third Parties’ Hostile Attribution Bias as the Boundary Condition

So far, we have proposed that negative affectivity plays a mediating role between peer abusive supervision and cyberloafing. Next, based on the AET, we will further explore the boundary condition of relationship between peer abusive supervision and third party’s negative affectivity. According to the AET (Weiss and Cropanzano, 1996), personal traits related to affective events at work will influence the individuals’ affective response and their cognitive judgment. Specific to the theoretical model of this study, as an unethical event, whether and how peer abusive supervision influence the negative affectivity of third-party employes depends on the third party’s sensitivity to other people’s mistreatment (Lin and Loi, 2019), because the understanding and response to unethical behavior varies from person to person (Alola et al., 2019). When the third party is more inclined to attribute the mistreatment of others to deliberate, the more likely he is to have negative affectivity after witnessing the abusive supervision. The hostile attribution bias can better reflect the above individual characteristics. Therefore, as an important trait related to affective cognition, hostile attribution bias can influence the affective response of negative affective work events (e.g., the peer abusive supervision) to the individual, which means that it may influence the relationship between peer abusive supervision and negative affectivity. Hostile attribution bias refers that individuals prefer attributing errors, responsibilities, and injuries to others, which is a type of external attribution tendency (De Castro et al., 2002). Individuals with high-level hostile attribution bias always think in a hostile way when analyzing the causes of work events (Chiu and Peng, 2008). Therefore, when a third party witnesses a colleague being abused, the observer with high-level hostile attribution bias is more inclined to attribute the supervisor’s abusive behavior to the supervisor, who is deliberately harming the colleague rather than urging the colleague to improve performance (Yu and Duffy, 2020). This will bring greater threat perception and challenges to third-party employes, which will correspondingly bring more negative affectivity. On the contrary, when a third party with low-level hostile attribution bias witnesses the abuse of colleagues by his supervisor, he is more likely to make cognitive judgments in a soft manner. Specifically, the third party may consider that the supervisor’s abusive supervision is just to urge colleagues to engage more in their work for performance improvement, which will bring less negative affectivity to third-party employes, as well.

In summary, we propose the following hypothesis:

*Hypothesis 5: Third party’s hostile attribution bias moderates the positive relationship between peer abusive supervision and third party’s negative affectivity such that the relationship is stronger when the third party’s hostile attribution bias is high.*

Combining hypothesis 4 (H4) and H5, the current study further proposes a moderated mediation hypothesis. Specifically, for third-party employes with high-level hostile attribution bias, they are more inclined to consider that their supervisor is deliberately harming colleagues after witnessing their colleagues being abused, which will bring greater threat perception, arousing their own negative affectivity. Therefore, the third parties will choose cyberloafing to stay away from negative affectivity. On the contrary, for the third parties with low-level hostile attribution bias, they may just consider that the supervisor is aimed at urging colleagues to work hard, which will be accompanied by less negative affectivity and cyberloafing behavior of the third party.

In summary, we propose the following hypothesis:

*Hypothesis 6: Third party’s hostile attribution bias moderates the indirect effect of peer abusive supervision on cyberloafing through negative affectivity such that this indirect relationship is stronger for third party with strong hostile attribution bias.*

## Method

### Sample and Procedures

First, the over-arching theory used in the current study is AET, an individual event level theory, which means we should verify the theoretical model at the individual level (Lin et al., 2021). Therefore, we should collect individual-level data as well. Second, different from other studies, the current study has two core variables: peer abusive supervision and cyberloafing. This puts forward several requirements for our target survey companies: (1) There exist supervisor-subordinate interaction in the company; (2) Employes have access to the Internet; and (3) Employes have high-level work autonomy so that they can engage in cyberloafing. Therefore, referring to previous literatures, we selected the service industry as our survey sample (Lim and Chen, 2012; Park and Kim, 2019). In particular, we collected the research data from 8 large service companies in southwest China. Third, since the present article studies the third parties affective and behavioral responses to the events their coworkers are experiencing, which means that event-related method should be employed during the survey process. Learning from previous literatures on peer abusive supervision from the third-party perspective (Priesemuth and Schminke, 2017), we used the critical incident technique method, which can effectively evaluate the employes’ perception and response to specific events (e.g., peer abusive supervision). Fourth, to better present the relationship between the various constructs and to avoid the influence of common method variance to the largest extent, we adopted a time-lag longitudinal tracking research design, which means that we have measured the variables at three different times. With reference to previous research, the interval between each survey is 2 weeks (Ding and Lin, 2020).

First, the investigators contacted large-scale service companies in southwest China (such as real estate, hotels, catering, finance, etc.). A total of 8 companies are willing to participate in the survey. Then we contacted the company’s human resources (HRs) specialists, and randomly selected 450 people as the participants based on the list of personnel provided by HR. Since our survey was conducted during the COVID-19 pandemic, in order to maintain social distancing, we created an electronic version of the questionnaire and completed the survey online in the form of email. Finally, since we conducted a longitudinal tracking survey, it means that we need to integrate the three-survey data to get the complete data. Therefore, for those samples that have failed to respond to a certain link, we will regard them as invalid responses and discard the samples. The specific process is as follows.

We sent out three emails to the participants in total, with an interval of 2 weeks between each email. In the first email, we explained the background and purpose of the survey, and informed the participants that they were voluntarily participated in the survey and their responses were anonymous, which would be used for this research only. Then, the participants were asked to recall the events, in which the supervisors are abusing their colleagues, they observed as much as possible. Finally, the participants reported their perceived peer abusive supervision and filled in the relevant background information. In the first email, 450 questionnaires were distributed, and 403 valid questionnaires were returned. Two weeks later, we sent the second email. The second survey mainly required the participants to report their negative affectivity. A total of 403 questionnaires were distributed and 378 were returned. Two weeks after the second survey, we conducted the third email distribution. This survey mainly required the participants to report their cyberloafing and hostile attribution bias. A total of 378 questionnaires were distributed and 355 were returned. The response rate of the entire survey was 78.9%. Please refer to Table 1 for the participants’ demographic information.

**TABLE 1:** Demographic information (*n* = 355).

Feature	Category	Quantity	Percentage
Gender	Male	147	41.4
	Female	208	58.6
Age	25 years old and below	49	13.8
	26–35 years old	96	27.0
	36–45 years old	175	49.3
	Over 46 years old	35	9.9
Education	Senior high school and below	27	7.6
	Training school	103	29.0
	Undergraduate	188	53.0
	Postgraduate and above	37	10.4
Tenure	0–2 years	162	45.6
	2–5 years	91	25.6
	5–10 years	73	20.6
	10 years and above	29	8.2

### Measures

#### Peer Abusive Supervision

In order to measure the peer abusive supervision perceived by third-party employes, we refer to the 5-item scale developed by Mitchell and Ambrose (2007), which is a simplified version of the Tepper (2000). The employes rate the supervisors’ behaviors frequency of abusing their colleagues (1 means never, 5 means always). One example item is: “My supervisor often taunts my colleague in front of others.” This scale has been widely used in previous studies (Zhang et al., 2020). The Cronbach’s alpha value of this scale is 0.899. Please refer to Table 2 for reliability and validity.

**TABLE 2:** Reliability and validity (*n* = 355).

	Factor loading	CR	AVE
Peer abusive supervision		0.928	0.720
PAS 1	0.753		
PAS 2	0.896		
PAS 3	0.904		
PAS 4	0.808		
PAS 5	0.873		
Negative affectivity		0.915	0.683
NA 1	0.757		
NA 2	0.800		
NA 3	0.800		
NA 4	0.892		
NA 5	0.876		
Cyberloafing		0.937	0.714
CL 1	0.891		
CL 2	0.907		
CL 3	0.835		
Hostile attribution bias		0.910	0.771
HAB 1	0.821		
HAB 2	0.865		
HAB 3	0.826		
HAB 4	0.821		
HAB 5	0.866		
HAB 6	0.868		

*“PAS” indicates peer abusive supervision, “NA” indicates third party’s negative affectivity, “CL” indicates third party’s cyberloafing, “HAB” indicates third party’s hostile attribution bias, CR indicates composite reliability, AVE indicates average variance extracted value.*

#### Third Party’s Negative Affectivity

We refer to the scale developed by Wong et al. (2006) and Wu et al. (2018) to measure the negative affectivity. There are 5 items. One example item is: “My work makes me unhappy.” The Cronbach’s alpha value of this scale is 0.883.

#### Third Party’s Hostile Attribution Bias

To measure the hostile attribution bias of the third party, we used the 6-item scale developed by Adams and John (1997). One example item is: “If the supervisor does not trust anyone, it will be a better choice.” The Cronbach’s alpha value of this scale is 0.850.

#### Third Party’s Cyberloafing

We used Lim and Teo (2005) 3-item scale to measure cyberloafing of the third party. Participants were asked to report the frequency of their engagement in cyberloafing behaviors using a scale ranging from “1 Never” to “5 Very Frequently.” This scale has been widely used in previous cyberloafing research (Karimi Mazidi et al., 2021). The Cronbach’s alpha value of this scale is 0.916.

#### Control Variables

In addition, studies have shown that some background variables of employes (such as age, gender, education, and tenure) are also important factors in the AET (Wegge et al., 2006; Cropanzano et al., 2017). Therefore, the above variables: employes’ gender (1 for male, 2 for female), age (coded from 1 to 4, representing 25 years old and below, 26–35 years old, 36–45 years old, and 46 and above, respectively), education (coded with 1 to 4, 1 means high school and below, 2 means college, 3 means undergraduate, and 4 means graduate and above, respectively) and tenure (1 means 2 years and below, 2 means 2–5 years, 3 means 5–10 years, and 4 means 10 years and above) are used as the control variables of this study and reported by the employes themselves.

### Analytic Strategy

Multiple methods were introduced to verify our theoretical framework model. First, we use OLS regression to test hypotheses 1, 2, 3, and 5, and further use PROCESS macro proposed by Preacher and Hayes (2008) to examine the mediation effect (hypothesis 4) and the moderated mediation effect (hypothesis 6). Specifically, we measure the difference in indirect effects between higher (+1 SD) and lower (−1 SD) level moderator variable (hostile attribution bias).

## Results

### Common Method Variance

In this study, time-lag method was carried out in the data collection stage, which can control the common method variance to a certain extent. However, our core variables involved in this article, peer abusive supervision, negative affectivity, cyberloafing, and hostile attribution bias are all evaluated by third-party employes, which may suffer the common method variance. Thus, the current study adopted the Harman’s single factor method to examine the common method variance. The percentage of first factor is 31.513% and the total is 71.204%. So, the above ratio is less than 50%, which means there is no common method variance in our data.

### Confirmatory Factor Analysis

We then performed confirmatory factor analysis (CFA) to test the variables’ discriminative validity (peer abusive supervision, negative affectivity, cyberloafing, and hostile attribution bias). The analysis results show that the four-factor model has the best fitting indicators, indicating that the variables have good discrimination validity. The results of the confirmatory factor analysis are shown in Table 3.

**TABLE 3:** Model fit results for confirmatory factor analyses (*n* = 355).

Model	χ^2^	df	CFI	RMR	RMSEA	Model comparison test		
						Model comparison	Δχ^2^	Δdf
1. Four factors::PAS; NA; CL; HAB	265.232	138	0.972	0.043	0.051			
2. Three factors a: PAS; NA + CL; HAB	1005.196	149	0.813	0.101	0.127	2 VS. 1	739.964	11
3. Three factors b: PAS + NA; CL; HAB	1323.630	149	0.743	0.096	0.149	3 VS. 1	1058.398	11
4. Three factors c: PAS + CL; NA; HAB	1089.936	149	0.794	0.124	0.134	4 VS. 1	824.704	11
5. Two factors: PAS + NA + CL; HAB	1783.354	151	0.643	0.127	0.175	5 VS. 1	1518.122	13
6. Single factor: PAS + NA + CL + HAB	2718.903	152	0.439	0.146	0.218	6 VS. 1	2253.671	14

*“PAS” indicates peer abusive supervision, “NA” indicates third party’s negative affectivity, “CL” indicates third party’s cyberloafing, “HAB” indicates third party’s hostile attribution bias; “+” indicates combination of factors; Δ, change relative to the measurement model; CFI, comparative fit index; TLI, Tucker-Lewis index; RMSEA, root mean squared error of approximation; RMR, root mean-square residual.*

### Correlation Analysis

According to the results shown in Table 4, there are positive correlated relationships between peer abusive supervision, third party’s negative affectivity, cyberloafing, and hostile attribution bias. In particular, peer abusive supervision was significantly positively correlated with the third party’s negative affectivity (*r* = 0.416, *p* < 0.01); peer abusive supervision was significantly positively correlated with the third party’s cyberloafing (*r* = 0.280, *p* < 0.01); and the third party’s negative affectivity was significantly positively correlated with his cyberloafing (*r* = 0.373, *p* < 0.01). Our correlation test results mean that we can proceed to the next regression test.

**TABLE 4:** Descriptive statistics and correlations (*n* = 355).

Variables	Mean	SD	1	2	3	4	5	6	7	8
Gender	1.59	0.49	1							
Age	3.55	0.85	0.062	1						
Education	2.67	0.77	–0.050	–0.034	1					
Tenure	1.91	0.99	−0.247**	0.198**	0.073	1				
PAS	1.81	0.73	–0.064	0.015	0.013	−0.229**	1			
NA	2.23	0.73	0.024	–0.033	0.059	−0.181**	0.416**	1		
CL	2.04	0.87	0.122*	–0.023	0.132*	−0.125*	0.280**	0.373**	1	
HAB	2.78	0.94	0.078	0.163**	−0.127*	0.210**	−0.316**	−0.315**	−0.432**	1

“PAS” indicates peer abusive supervision, “NA” indicates third party’s negative affectivity, “CL” indicates third party’s cyberloafing, “HAB” indicates third party’s hostile attribution bias; *p < 0.05, **p < 0.01.

### Hypothesis Testing

We will then conduct a series of regression analysis to test our theoretical hypotheses.

First, we will test the main effect of the thesis, that is, whether the positive influence of peer abusive supervision on third parties’ cyberloafing is significant. Model 5 in Table 5 is the regression result of this hypothesis. As shown in the attached table, after adding the control variables, peer abusive supervision can still significantly negatively predict the third parties’ cyberloafing (β = 0.280, *p* < 0.01). So far, Hypothesis 1 has been verified.

**TABLE 5:** Regression results for the predictors of third party’s cyberloafing (*n* = 355).

Variables	Third party’s cyberloafing (T3)											
	Model 4			Model 5			Model 6			Model 7		
	*b*	*SE*	*t*	*b*	*SE*	*t*	*b*	*SE*	*t*	*b*	*SE*	*t*
Third party’s gender (T1)	0.208	0.110	1.886^†^	0.282	0.107	2.638**	0.224	0.103	2.172*	0.264	0.103	2.571*
Third party’s age (T1)	0.000	0.063	–0.003	–0.026	0.061	–0.425	–0.005	0.059	–0.079	–0.019	0.059	–0.322
Third party’s education (T1)	0.190	0.069	2.769**	0.178	0.066	2.699**	0.156	0.064	2.430*	0.155	0.064	2.444*
Third party’s tenure (T1)	–0.111	0.056	−1.983*	–0.033	0.056	–0.589	–0.041	0.053	–0.777	–0.008	0.054	–0.152
Peer abusive supervision (T1)				0.280**	0.052	5.363**				0.163	0.054	2.990**
Third party’s negative affectivity (T2)							0.356	0.050	7.141**	0.293	0.054	5.473**
Constant	–0.622	0.348	−1.787^†^	–0.766	0.336	−2.281*	–0.675	0.326	−2.073*	–0.750	0.323	−2.322*
*R* ^2^		0.045			0.118			0.167			0.188	
ΔR^2^					0.073			0.122			0.143	
*F*		4.161**			9.346**			14.004**			13.426**	

T1/2/3 = Time 1/2/3; unstandardized regression coefficients are reported; ^†^p < 0.10, *p < 0.05, **p < 0.01.

Second, we will test the mediating effect in the model. Specially, we will examine the mediating role of third parties’ negative affectivity among the relationship between peer abusive supervision and third parties’ cyberloafing. The model 1 in Table 6 is the regression result of the control variables on third parties’ negative affectivity, and the model 2 is the regression result after adding peer abusive supervision. As shown in model 2, peer abusive supervision positively predicts the third parties’ negative affectivity (β = 0.399, *p* < 0.05). So far, Hypothesis 2 has been verified. Model 6 in Table 5 is the regression result of third parties’ negative affectivity on their cyberloafing. As shown in model 6, the third parties’ negative affectivity is positively predicting their cyberloafing (β = 0.356, *p* < 0.01). So far, Hypothesis 3 has been verified. Model 7 is the regression result of the dependent variable (third parties’ negative affectivity) by putting the independent variable (peer abusive supervision) and the mediating variable (third parties’ negative affectivity) into the equation at the same time. As shown in model 7, the predictive effects of peer abusive supervision (β = 0.163, *p* < 0.01) and third parties’ negative affectivity (β = 0.293, *p* < 0.01) on their cyberloafing are both significant. At the same time, the predictive value of peer abusive supervision on third parties’ cyberloafing is relatively lower. Therefore, Hypothesis 4 has been initially verified. At last, following the previous studies, we further examine the mediating effect according to the bootstrap method. Please refer to Table 7 for specific examination results. As shown in Table 7, the indirect impact of peer abusive supervision on third parties’ cyberloafing *via* third parties’ negative affectivity is significant (index = 0.1169, 95% CI [0.0491, 0.1647]). Therefore, Hypotheses 2, 3, and 4 have been verified again.

**TABLE 6:** Regression results for the predictors of third party’s negative affectivity (*n* = 355).

Variables	Third party’s negative affectivity (T2)								
	Model 1			Model 2			Model 3		
	*b*	*SE*	*t*	*b*	*SE*	*t*	*b*	*SE*	*t*
Third party’s gender (T1)	–0.045	0.110	–0.410	0.061	0.103	0.591	0.075	0.101	0.744
Third party’s age (T1)	0.013	0.063	0.199	–0.024	0.059	–0.410	–0.009	0.058	–0.151
Third party’s education (T1)	0.094	0.069	1.372	0.078	0.063	1.227	0.038	0.062	0.610
Third party’s tenure (T1)	–0.195	0.056	−3.482**	–0.084	0.053	–1.573	–0.036	0.053	–0.680
Peer abusive supervision (T1)				0.399	0.050	7.958**	0.353	0.051	6.944**
Third party’s hostile attribution bias (T3)							–0.208	0.052	−4.040**
Peer abusive supervision (T1) X Hostile attribution bias (T3)							0.108	0.042	2.597*
Constant	0.149	0.349	0.428	–0.057	0.323	0.428	–0.086	0.318	–0.271
*R* ^2^		0.038			0.186			0.217	
ΔR^2^					0.148			0.194	
*F*		3.494**			15.958**			15.046**	

T1/2/3 = Time 1/2/3; unstandardized regression coefficients are reported; *p < 0.05, **p < 0.01.

**TABLE 7:** Bootstrap results for the mediation effect (*n* = 355).

Direct impact of peer abusive supervision on third party’s cyberloafing					
Effect	S.E.	T	p	LLCI	ULCI
0.1630	0.0545	2.9901	0.0030	0.0558	0.2701

**Indirect impact of peer abusive supervision on third party’s cyberloafing**					

	Effect	Boot SE	Boot LLCI	Boot ULCI	
Negative affectivity	0.1169	0.0287	0.0654	0.1793	

*LLCI and ULCI indicate the minimum and maximum values of the CI; this study uses bootstrap for random sampling 5000 times.*

**FIGURE 2:**
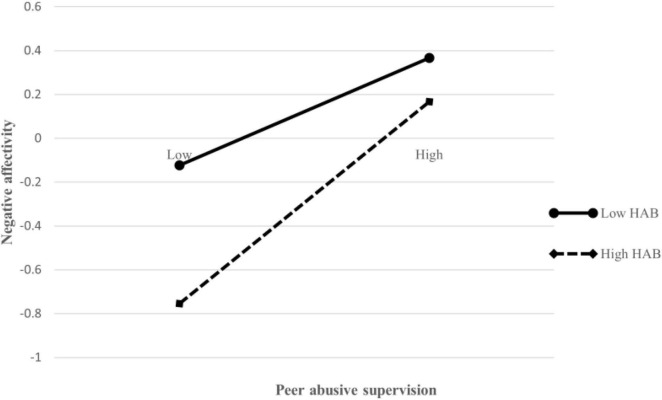
The moderating role of third party’s hostile attribution bias on the relationship between peer abusive supervision and third party’s negative affectivity.

Next, we will test the moderating effect. We first constructed the interaction term between the independent variable (peer abusive supervision) and the moderating variable (third parties’ hostile attribution bias), and then we put it into the regression equation. As model 3 in Table 6 shown, the interaction item negatively predicts the third parties’ negative affectivity (β = 0.108, *p* < 0.05). In order to present the above adjustment effect more intuitively, we have further drawn a simple slope diagram. As shown in Figure 2, the higher the hostile attribution bias of third parties, the stronger the positive impact of peer abusive supervision on third parties’ negative affectivity. Therefore, Hypothesis 5 is verified.

**FIGURE 3:**
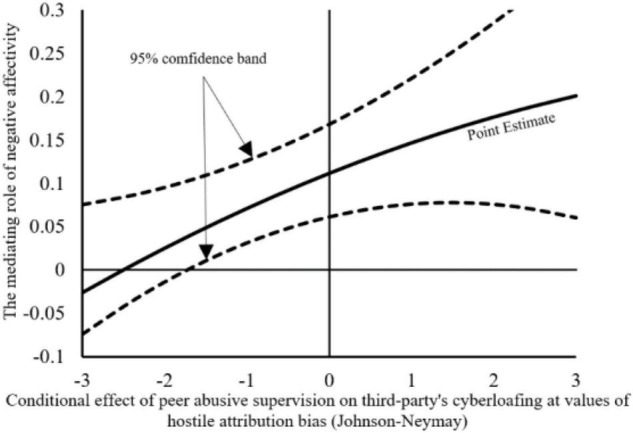
Conditional effect of peer abusive supervision on third party’s cyberloafing at values of third party’s hostile attribution bias.

Finally, we will conduct a moderated mediation effect test, which will verify whether the third parties’ hostile attribution bias moderates the mediation effect of third parties’ negative affectivity. As shown in Table 8, when the third-party employes have high hostile attribution bias, the above indirect effect is significant (*b* = 0.1443, 95%CI = [0.0756, 0.2170]). When the hostile attribution bias of third-party employes is low, the above indirect effect is significant (*b* = 0.0713, 95% CI = [0.0319, 0.1263]). The difference between the above two model is significant (Δb = 0.0317, 95% CI = [0.0021, 0.0586]). Therefore, H6 is verified (refer to Edwards and Lambert (2007) mediated interaction effect drawing method). Hypothesis 6 corresponds to the moderated mediation effect diagram shown in Figure 3.

**TABLE 8:** Bootstrap results for the moderated mediation effect (*n* = 355).

	Conditional indirect effect				Moderated mediator			
	Estimate	Boot SE	BC 95% CI		INDEX	S.E.	BC 95% CI	
Low HAB	0.0713	0.0240	0.0319	0.1263	0.0317	0.0139	0.0021	0.0586
Middle HAB	0.1104	0.0269	0.0608	0.1667				
High HAB	0.1443	0.0364	0.0756	0.2170				

*HAB indicates hostile attribution bias, low HAB represents mean “−1” SD, and high HAB represents mean “+1” SD; BC indicates biased corrected. This study uses bootstrap for random sampling 5000 times.*

## Discussion

Drawing on the AET, the current study constructed an affective process model for third party’s cyberloafing reaction to peer abusive supervision, which helps to explain the affective mechanism during the cyberloafing process and the boundary conditions of the above “event-affectivity-behavior” framework. Specifically, we suggest that peer abusive supervision constitutes the negative affective event for the third-party employes, which will bring uncertainty and threat perception to the third-party employes and further cause their negative affectivity. Therefore, the third party employes often choose cyberloafing to stay away from this negative state. In addition, the third-party employes with high-level hostile attribution bias tend to attribute the peer abusive supervision they witnessed to the supervisor’s malicious motives, resulting in higher levels of negative affectivity and more cyberloafing. These research results provide theoretical and interventional enlightenment for inhibiting cyberloafing from the perspective of third party employes.

### Theoretical Implications

The current study has important theoretical implications for the research of abusive supervision and cyberloafing, as well as the AET.

First, this study explored the impact of abusive supervision on third-party employes based on the perspective of event observers, which enriches the research perspective of abusive supervision. Previous literatures on abusive supervision mainly explored its impact on the employes being abused from the perspective of victims. For example, abusive supervision may increase an employe’s emotional exhaustion, work dissatisfaction, reduce his/her work engagement and task performance, and even his/her CWB (Tepper, 2000; Mitchell and Ambrose, 2007; Harris et al., 2013; Fischer et al., 2021). However, the abusive supervision event involves three parties: the supervisor (behavior perpetrator), the abused employe (behavior receiver), and the third party (behavior observer), which means that the previous literatures have ignored the role of third party. By shifting the focus on observer, the current study emphasizes that peer abusive supervision, a negative affective event, can bring threat perceptions and induce negative affectivity to third-party employes, who will further stay away from this kind of negative state through cyberloafing. Our work reveals the impact of abusive supervision on another important group in the workplace, which is not only a useful supplement to the research on abusive supervision from a third-party perspective, but also a positive response to previous scholars’ calls for research from multiple perspectives (Harris et al., 2013; Tepper et al., 2017). This perspective has certain practical significance, because although the impact of abusive supervision on third parties is an indirect effect, in reality, it is often a “majority effect,” that is, usually the third party is the majority, and the behavior recipient (employes being abused) may be the minority (Zhou et al., 2020). In short, our theoretical model has certain reference significance for understanding whether, how, and when peer abusive supervision will bring negative impacts to the third party.

Second, the present study found new antecedent variables that affect employe cyberloafing. So far, previous studies have conducted large discussions on the antecedents of cyberloafing. Previous literatures have conducted rich investigation on the driving factors of cyberloafing. For example, sociodemographics, personality traits, emotion, habits, organizational infrastructure, organizational culture, monitoring strategies, and other factors have been regarded as the antecedent variables of cyberloafing (Weissenfeld et al., 2019; Lim et al., 2021; Usman et al., 2021). That is to say, the past literatures mostly focused on the individual and organization factors, failing to integrate the supervisory factor into the research of employe cyberloafing (Kim et al., 2016), especially ignoring to study the impact of supervisor on the largest group (event observers) in the organization. Our research shows that when the third-party employes witness their colleagues being abused, they will have negative affectivity and will choose cyberloafing to stay away from this negative state. Combined with previous research (Agarwal Upasnaa and Avey James, 2020), abusive supervision not only has a large negative impact on the physical and mental health of victims, but also influences the affective state of bystanders that may result in most members of the organization to implement deviant behaviors, which is harmful to team development (Robinson and Bennett, 1995). This result shows that, in addition to individual and organization factors, the events observed by employes can also cause their cyberloafing.

Third, this study enriches the application of AET and contributes to the theoretical boundary of this theory. On the one hand, this study expanded the application of AET from the perspective of third party. The AET, which links employe emotions, attitudes, and behaviors together in the organization research, has been applied to the development and empirical research of some new theories (Ferris et al., 2008; Rodell and Judge, 2009). On this basis, drawing on AET’s over-arching framework, this study constructs the theoretical model of abusive supervision influencing third-party employes’ cyberloafing, and their negative affectivity as the mediating variable among the above relationships, which provides empirical support for the theoretical relationship among work events, third parties work attitudes, and their behaviors in the AET. Unlike the previous empirical studies using behavior recipient perspective (Matta et al., 2014; Todorova et al., 2014), the current study explores the impact of work events on employes’ affectivity and work from the behavioral observers’ perspective, which helps to expand the application of AET. In addition, we have further expanded its theoretical boundaries based on the AET. Based on the AET, we found that the third parties hostile attribution bias will moderate the effect of peer abusive supervision on their negative affectivity. Specially, for employes with high-level hostile attribution bias, the negative impact of peer abusive supervision on third-party employes is more serious. Correspondingly, for employes with low-level hostile attribution bias, the negative impact of peer abusive supervision on third-party employes is relatively small. Therefore, hostile attribution bias moderates the impact of the affective events on third parties. From this perspective, the hostile attribution bias of third-party employes may be a theoretical boundary of affective events influencing their own cognition and affectivity.

### Practical Implications

Our research results also provide a useful reference for the improvement of management practices in the workplace. First, our framework shows that abusive supervision will not only negatively influence the victims, but also negatively affect the observers of the event. Specifically, peer abusive supervision will cause negative affectivity to observers, and third-party employes will punish supervisors through negative behaviors such as cyberloafing, which is obviously not conducive to the sustainable development of the organization. Therefore, it is necessary for the organization to carry out relevant management skills training to supervisors to improve their awareness of the harmfulness of abusive supervision, which may help to minimize the possibility of abusive supervision.

Second, the results of this article show that individuals with high-level hostile attribution bias may be more inclined to attribute the supervisor’s behavior to their colleagues, which they witnessed as the supervisor’s malicious motives, and thus will bring them negative affectivity and the subsequent cyberloafing behavior. This personality trait may cause employes to misunderstand some of the supervisor’s behavior, thereby affecting their subsequent work commitment and overall team performance. Therefore, the company can add a test for the personality traits of employes when recruiting. For those candidates with extremely hostile attribution bias, the company should carefully consider whether to hire such employes to avoid the trouble that they may bring to the team.

Third, this research shows that when the third-party employes witness the peer abusive supervision, they will choose cyberloafing to punish their supervisors. However, one thing that needs to be pointed out is that cyberloafing is a kind of counterwork behavior, which causes great harm to the team (Wagner et al., 2012). However, as we explained in the theory section, cyberloafing is very concealed and not easy to find. Therefore, there will be more and more employes engaged in cyberloafing for it is not easy to be detected by the supervisor, which may bring great potential harm to the team. Therefore, the organization should take action to curb this negative behavior to avoid secondary harm caused by abusive supervision. For example, the organizations can provide employes with professional ethics training or monitor computer screens to reduce cyberloafing.

### Limitations and Future Research

The current study explores the harm of peer abusive supervision to observers from a third-party perspective, which is innovative. Although this study has many advantages, there are still some shortcomings, which also provide some opportunities for further research. First, the data collected in this article come from 8 large service-oriented companies in southwest China. Future research can try to collect samples from other industries to further verify the universal adaptability value of the conclusions of this article.

Second, the variables in this article are reported by a single source of third-party employes, which often leads to common method variance (Podsakoff et al., 2012). In order to solve the above problem, we designed a longitudinal survey measuring the core variables at 3 times. Furthermore, we used a single-factor method to perform a common method variance test, and the results meet the requirements, which indicate that our sample data do not have a serious common method variance (CMV) problem, and follow-up statistical tests can be performed. Future research may consider using Fornell Larcker test or heterotrait-monotrait ratio (HTMT) for examination again, which is more robust and credible. Meanwhile, scholars can try to collect data from multiple sources and use multilevel regression (such as hierarchical linear regression) to verify the theoretical model of this article again to improve the robustness of the results.

Third, the present research explores the negative affectivity of third-party employes as the mediating mechanism connecting negative workplace events and employe behavioral responses. However, drawing on AET, negative affectivity to negative events can influence employes’ negative behaviors through two paths (Weiss and Cropanzano, 1996). The first path is the direct influence path of affectivity on employe behaviors: Affect-Driven Behaviors; the second path is through influencing individual cognitive judgments, and affectivity indirectly affects employe behaviors: Judgment-Driven Behaviors. This means that there may be a third-party employes’ cognitive mechanism between negative affectivity and their negative behavioral responses, so future research can further explore this topic.

Fourth, this study is based on the AET, and it explores only the mediating role of negative affectivity between peer abusive supervision and third-party employe’s cyberloafing. Follow-up research may consider other mediation paths. For example, peer abusive supervision may influence the psychological safety of third-party employes, which in turn affects their subsequent behavior.

## Conclusion

Drawing on the AET, the current study constructed a theoretical framework of third party’s cyberloafing reactions to peer abusive supervision, which helps to explain the affective mechanism and boundary conditions of the above “events-affectivity -behavior” model. Based on a multiwave data from 355 service-oriented employes, we found that peer abusive supervision has a significant positive impact on the third party’s cyberloafing; the third party’s negative affectivity plays a mediating role in these relationships. In addition, the third party’s hostile attribution bias moderates the mediating role of his/her negative affectivity. Specifically, the higher the hostile attribution bias, the greater the mediating role of negative affectivity.

## Data Availability Statement

The data used in this study are available upon request to the corresponding author.

## Ethics Statement

Ethical review and approval was not required for the current study in accordance with the local legislation and institutional requirements. Written informed consent for participation was not required for this study in accordance with the local legislation and the institutional requirements.

## Author Contributions

XL contributed to conceptualization, data curation, and original draft. GG contributed to conceptualization, methodology, original draft, and formal analysis. QG contributed to methodology and original draft. SL contributed to methodology and formal analysis. ZL contributed to data curation, visualization, and funding acquisition. All authors contributed to the article and approved the submitted version.

## Conflict of Interest

The authors declare that the research was conducted in the absence of any commercial or financial relationships that could be construed as a potential conflict of interest.

## Publisher’s Note

All claims expressed in this article are solely those of the authors and do not necessarily represent those of their affiliated organizations, or those of the publisher, the editors and the reviewers. Any product that may be evaluated in this article, or claim that may be made by its manufacturer, is not guaranteed or endorsed by the publisher.

## References

[B1] Adams S. H. John O. P. (1997). A hostility scale for the California psychological inventory: MMPI, observer Q-sort, and Big-five correlates. *J. Pers. Assess.* 69 408–424. 10.1207/s15327752jpa6902_11 9392898

[B2] Agarwal U. A. Avey J. B. (2020). Abusive supervisors and employees who cyberloaf: examining the roles of psychological capital and contract breach. *Int. Res.* 30 789–809. 10.1108/INTR-05-2019-0208

[B3] Agarwal Upasnaa A. Avey James B. (2020). Abusive supervisors and employees who cyberloaf: examining the roles of psychological capital and contract breach. *Int. Res.* 30 789–809. 10.1108/INTR-05-2019-0208

[B4] Alola U. V. Olugbade O. A. Avci T. Öztüren A. (2019). Customer incivility and employees’ outcomes in the hotel: testing the mediating role of emotional exhaustion. *Tour. Manag. Persp.* 29 9–17. 10.1016/j.tmp.2018.10.004

[B5] Ashkanasy N. M. Ayoko O. B. Jehn K. A. (2014). Understanding the physical environment of work and employee behavior: an affective events perspective. *J. Organ. Behav.* 35 1169–1184. 10.1002/job.1973

[B6] Askew K. Buckner J. E. Taing M. U. Ilie A. Bauer J. A. Coovert M. D. (2014). Explaining cyberloafing: the role of the theory of planned behavior. *Comp. Hum. Behav.* 36 510–519. 10.1016/j.chb.2014.04.006

[B7] Askew K. L. Buckner J. E. (2017). The role of the work station: visibility of one’s computer screen to coworkers influences cyberloafing through self-efficacy to hide cyberloafing. *Psychol. Manag. J.* 20 267–287. 10.1037/mgr0000061

[B8] Batabyal S. Bhal K. (2020). Traditional cyberloafing, mobile cyberloafing and personal mobile-internet loafing in business organizations: exploring cognitive ethical logics. *J. Inform. Commun. Ethics Soc.* 18, 631–647. 10.1108/JICES-07-2019-0081

[B9] Baturay M. H. Toker S. (2015). An investigation of the impact of demographics on cyberloafing from an educational setting angle. *Comp. Hum. Behav.* 50 358–366. 10.1016/j.chb.2015.03.081

[B10] Bono J. Glomb T. Shen W. Kim E. Koch A. (2012). Building positive resources: effects of positive events and positive reflection on work stress and health. *Acad. Manag. J.* 56 1601–1627. 10.5465/amj.2011.0272

[B11] Chen C. Qin X. Yam K. C. Wang H. (2020). Empathy or schadenfreude? Exploring observers’ differential responses to abusive supervision. *J. Bus. Psychol.* 36 1077–1094. 10.1007/s10869-020-09721-4

[B12] Cheng B. Zhou X. Guo G. Yang K. (2020). Perceived Overqualification and cyberloafing: a moderated-mediation model based on equity theory. *J. Bus. Ethics* 164 565–577. 10.1007/s10551-018-4026-8

[B13] Chiu S.-F. Peng J.-C. (2008). The relationship between psychological contract breach and employee deviance: the moderating role of hostile attributional style. *J. Vocat. Behav.* 73 426–433. 10.1016/j.jvb.2008.08.006

[B14] Colquitt J. Conlon D. Wesson M. Porter C. Ng K. (2001). Justice at the millennium: a meta-analytic review of 25 years of organizational justice research. *J. Appl. Psychol.* 86 425–445. 10.1037/0021-9010.86.3.425 11419803

[B15] Cropanzano R. Dasborough M. T. Weiss H. M. (2017). Affective events and the development of leader-member exchange. *Acad. Manag. Rev.* 42 233–258. 10.5465/amr.2014.0384

[B16] Dasborough M. T. (2006). Cognitive asymmetry in employee emotional reactions to leadership behaviors. *Leadersh. Q.* 17 163–178. 10.1016/j.leaqua.2005.12.004

[B17] De Castro B. O. Veerman J. W. Koops W. Bosch J. D. Monshouwer H. J. (2002). Hostile attribution of intent and aggressive behavior: a meta-analysis. *Child Dev.* 73 916–934. 10.1111/1467-8624.00447 12038560

[B18] Dhanani L. Y. Lapalme M. L. (2019). It’s not personal: a review and theoretical integration of research on vicarious workplace mistreatment. *J. Manag.* 45 2322–2351. 10.1177/0149206318816162

[B19] Dimotakis N. Scott B. Koopman J. (2011). An experience sampling investigation of workplace interactions, affective states, and employee well-being. *J. Organ. Behav.* 32 572–588. 10.1002/job.722

[B20] Ding H. Lin X. (2020). Individual-focused transformational leadership and employee strengths use: the roles of positive affect and core self-evaluation. *Person. Rev.* 50 1022–1037. 10.1108/PR-10-2019-0541

[B21] Edwards J. R. Lambert L. S. (2007). Methods for integrating moderation and mediation: a general analytical framework using moderated path analysis. *Psychol. Methods* 12 1–22. 10.1037/1082-989X.12.1.1 17402809

[B22] Einarsen S. Aasland M. S. Skogstad A. (2007). Destructive leadership behaviour: a definition and conceptual model. *Leadersh. Q.* 18 207–216. 10.1016/j.leaqua.2007.03.002

[B23] Ferris G. R. Munyon T. P. Basik K. Buckley M. R. (2008). The performance evaluation context: social, emotional, cognitive, political, and relationship components. *Hum. Resour. Manag. Rev.* 18 146–163. 10.1016/j.hrmr.2008.07.006

[B24] Fischer T. Tian A. W. Lee A. Hughes D. J. (2021). Abusive supervision: a systematic review and fundamental rethink. *Leadersh. Q.* 32:101540. 10.1016/j.leaqua.2021.101540

[B25] Glasø L. Vie T. L. Holmdal G. R. Einarsen S. (2011). An application of affective events theory to workplace bullying: the role of emotions, trait anxiety, and trait anger. *Eur. Psychol.* 16 198–208. 10.1027/1016-9040/a000026

[B26] Grandey A. Tam A. Brauburger A. (2002). Affective states and traits in the workplace: diary and survey data from young workers. *Motiv. Emot.* 26 31–55. 10.1023/A:1015142124306

[B27] Harris K. J. Harvey P. Harris R. B. Cast M. (2013). An investigation of abusive supervision, vicarious abusive supervision, and their joint impacts. *The J. Soc. Psychol.* 153 38–50. 10.1080/00224545.2012.703709 23421004

[B28] Hensel P. G. Kacprzak A. (2020). Job overload, organizational commitment, and motivation as antecedents of cyberloafing: evidence from employee monitoring software. *Eur. Manag. Rev.* 17 931–942. 10.1111/emre.12407

[B29] Hensel P. G. Kacprzak A. (2021). Curbing cyberloafing: studying general and specific deterrence effects with field evidence. *Eur. J. Inform. Syst.* 30 219–235. 10.1080/0960085X.2020.1756701

[B30] Jo Ann O. (2019). “Cyberloafing and constructive recreation,” in *Advanced Methodologies and Technologies in Business Operations and Management*, ed. Mehdi Khosrow-Pour D. B. A. (Hershey, PA: IGI Global).

[B31] Judge T. Scott B. Ilies R. (2006). Hostility, job attitudes, and workplace deviance: test of a multilevel model. *J. Appl. Psychol.* 91 126–138. 10.1037/0021-9010.91.1.126 16435943

[B32] Karimi Mazidi A. Rahimnia F. Mortazavi S. Lagzian M. (2021). Cyberloafing in public sector of developing countries: job embeddedness as a context. *Person. Rev.* 50, 1705–1738. 10.1108/PR-01-2020-0026

[B33] Khansa L. Barkhi R. Ray S. Davis Z. (2018). Cyberloafing in the workplace: mitigation tactics and their impact on individuals’ behavior. *Inform. Technol. Manag.* 19 197–215. 10.1007/s10799-017-0280-1

[B34] Khansa L. Kuem J. Siponen M. Kim S. S. (2017). To cyberloaf or not to cyberloaf: the impact of the announcement of formal organizational controls. *J. Manag. Inform. Syst.* 34 141–176. 10.1080/07421222.2017.1297173

[B35] Kim K. Del Carmen Triana M. Chung K. Oh N. (2016). When do employees cyberloaf? An interactionist perspective examining personality, justice, and empowerment. *Hum. Resour. Manag.* 55 1041–1058. 10.1002/hrm.21699

[B36] Koay Kian Y. (2018). Workplace ostracism and cyberloafing: a moderated–mediation model. *Int. Res.* 28 1122–1141. 10.1108/IntR-07-2017-0268

[B37] Landers R. N. Callan R. C. (2014). Validation of the beneficial and harmful work-related social media behavioral taxonomies: development of the work-related social media questionnaire. *Soc. Sci. Comp. Rev.* 32 628–646. 10.1177/0894439314524891

[B38] Lim P. K. Koay K. Y. Chong W. Y. (2021). The effects of abusive supervision, emotional exhaustion and organizational commitment on cyberloafing: a moderated-mediation examination. *Int. Res.* 31 497–518. 10.1108/INTR-03-2020-0165

[B39] Lim V. (2002). The IT way of loafing on the job: cyberloafing, neutralizing and organizational justice. *J. Organ. Behav.* 23 675–694. 10.1002/job.161

[B40] Lim V. K. G. Chen D. J. Q. (2012). Cyberloafing at the workplace: gain or drain on work? *Behav. Inform. Technol.* 31 343–353. 10.1080/01449290903353054

[B41] Lim V. K. G. Teo T. S. H. (2005). Prevalence, perceived seriousness, justification and regulation of cyberloafing in Singapore: an exploratory study. *Inform. Manag.* 42 1081–1093. 10.1016/j.im.2004.12.002

[B42] Lin S.-H. Chang C.-H. Lee H. W. Johnson R. E. (2021). Positive family events facilitate effective leader behaviors at work: a within-individual investigation of family-work enrichment. *J. Appl. Psychol.* 106, 1412–1434. 10.1037/apl0000827 32969705

[B43] Lin X. Loi R. (2019). Punishing the perpetrator of incivility: the differential roles of moral identity and moral thinking orientation. *J. Manag.* 47 898–929. 10.1177/0149206319870236

[B44] Matta F. K. Erol-Korkmaz H. T. Johnson R. E. Biçaksiz P. (2014). Significant work events and counterproductive work behavior: the role of fairness, emotions, and emotion regulation. *J. Organ. Behav.* 35 920–944. 10.1002/job.1934

[B45] Matthews B. A. Norris F. H. (2002). When is believing “seeing”? Hostile attribution bias as a function of self-reported aggression1. *J. Appl. Soc. Psychol.* 32 1–31. 10.1111/j.1559-1816.2002.tb01418.x

[B46] Mei M. Yang F. Tang M. (2021). Does practice enhance adaptability? the role of personality trait, supervisor behavior, and career development training. *Front. Psychol.* 11:594791. 10.3389/fpsyg.2020.594791 33613355 PMC7890024

[B47] Mitchell M. S. Ambrose M. L. (2007). Abusive supervision and workplace deviance and the moderating effects of negative reciprocity beliefs. *J. Appl. Psychol.* 92 1159–1168. 10.1037/0021-9010.92.4.1159 17638473

[B48] Mitchell M. S. Vogel R. M. Folger R. (2015). Third parties’ reactions to the abusive supervision of coworkers. *J. Appl. Psychol.* 100 1040–1055. 10.1037/apl0000002 25243999

[B49] Mor N. Winquist J. (2002). Self-focused attention and negative affect: a meta-analysis. *Psychol. Bull.* 128 638–662. 10.1037/0033-2909.128.4.638 12081086

[B50] O’Neill T. A. Hambley L. A. Bercovich A. (2014a). Prediction of cyberslacking when employees are working away from the office. *Comput. Hum. Behav.* 34 291–298. 10.1016/j.chb.2014.02.015

[B51] O’Neill T. A. Hambley L. A. Chatellier G. S. (2014b). Cyberslacking, engagement, and personality in distributed work environments. *Comput. Hum. Behav.* 40 152–160. 10.1016/j.chb.2014.08.005

[B52] O’Reilly J. Aquino K. (2011). A model of third parties’ morally motivated responses to mistreatment in organizations. *Acad. Manag. Rev.* 36 526–543. 10.5465/AMR.2011.61031810

[B53] Ouyang K. Lam W. Wang W. (2015). Roles of gender and identification on abusive supervision and proactive behavior. *Asia Pac. J. Manag.* 32 671–691. 10.1007/s10490-015-9410-7

[B54] Park J. Kim H. J. (2019). How and when does abusive supervision affect hospitality employees’ service sabotage? *Int. J. Hosp. Manag.* 83 190–197. 10.1016/j.ijhm.2018.10.014

[B55] Peng A. C. Schaubroeck J. M. Li Y. (2013). Social exchange implications of own and coworkers’ experiences of supervisory abuse. *Acad. Manag. J.* 57 1385–1405. 10.5465/amj.2012.0080

[B56] Podsakoff P. M. Mackenzie S. B. Podsakoff N. P. (2012). Sources of method bias in social science research and recommendations on how to control it. *Annu. Rev. Psychol.* 63 539–569. 10.1146/annurev-psych-120710-100452 21838546

[B57] Preacher K. J. Hayes A. F. (2008). Asymptotic and resampling strategies for assessing and comparing indirect effects in multiple mediator models. *Behav. Res. Methods* 40 879–891. 10.3758/BRM.40.3.879 18697684

[B58] Priesemuth M. Schminke M. (2017). Helping thy neighbor? Prosocial reactions to observed abusive supervision in the workplace. *J. Manag.* 45 1225–1251. 10.1177/0149206317702219

[B59] Reich T. C. Hershcovis M. S. (2015). Observing workplace incivility. *J. Appl. Psychol.* 100 203–215. 10.1037/a0036464 24731181

[B60] Robinson S. L. Bennett R. J. (1995). A typology of deviant workplace behaviors: a multidimensional scaling study. *Acad. Manag. J.* 38 555–572. 10.5465/256693

[B61] Rodell J. B. Judge T. A. (2009). Can “good” stressors spark “bad” behaviors? The mediating role of emotions in links of challenge and hindrance stressors with citizenship and counterproductive behaviors. *J. Appl. Psychol.* 94 1438–1451. 10.1037/a0016752 19916654

[B62] Salary.com (2014). *Wasting Time at Work.* Waltham, MA: Salary.com.

[B63] Seo M.-G. Barrett L. Bartunek J. (2004). The role of affective experience in work motivation. *Acad. Manag. Rev.* 29 423–439. 10.2307/20159052 PMC1519413 16871321

[B64] Shao P. Li A. Mawritz M. (2018). Self-protective reactions to peer abusive supervision: the moderating role of prevention focus and the mediating role of performance instrumentality. *J. Organ. Behav.* 39 12–25. 10.1002/job.2206

[B65] Shao Y. D. Fang Y. R. Wang M. Chang C. H. Wang L. (2021). Making daily decisions to work from home or to work in the office: the impacts of daily work- and covid-related stressors on next-day work location. *J. Appl. Psychol.* 106 825–838. 10.1037/apl0000929 34138589

[B66] Tandon A. Kaur P. Ruparel N. Islam J. U. Dhir A. (2021). Cyberloafing and cyberslacking in the workplace: systematic literature review of past achievements and future promises. *Int. Res.* (in press). 10.1108/INTR-06-2020-0332

[B67] Tepper B. (2000). Consequences of abusive supervision. *Acad. Manag. J.* 43 178–190. 10.5465/1556375

[B68] Tepper B. J. Simon L. Park H. M. (2017). Abusive supervision. *Annu. Rev. Organ. Psychol. Organ. Behav.* 4 123–152. 10.1146/annurev-orgpsych-041015-062539

[B69] Thoresen C. J. Kaplan S. A. Barsky A. P. Warren C. R. De Chermont K. (2003). The affective underpinnings of job perceptions and attitudes: a meta-analytic review and integration. *Psychol. Bull.* 129 914–945. 10.1037/0033-2909.129.6.914 14599288

[B70] Todorova G. Bear J. B. Weingart L. R. (2014). Can conflict be energizing? A study of task conflict, positive emotions, and job satisfaction. *J. Appl. Psychol.* 99 451–467. 10.1037/a0035134 24295533

[B71] Usman M. Javed U. Shoukat A. Bashir N. A. (2021). Does meaningful work reduce cyberloafing? Important roles of affective commitment and leader-member exchange. *Behav. Inform. Technol.* 40 206–220. 10.1080/0144929X.2019.1683607

[B72] Wagner D. Barnes C. Lim V. Ferris D. (2012). Lost sleep and cyberloafing: evidence from the laboratory and a daylight saving time quasi-experiment. *J. Appl. Psychol.* 97 1068–1076. 10.1037/a0027557 22369272

[B73] Wang H. Li Y. (2019). Role overload and Chinese nurses’ satisfaction with work-family balance: the role of negative emotions and core self-evaluations. *Curr. Psychol.* 40, 5515–5525. 10.1007/s12144-019-00494-5

[B74] Wegge J. Dick R. V. Fisher G. K. West M. A. Dawson J. F. (2006). A test of basic assumptions of affective events theory (Aet) in call centre work1. *Br. J. Manag.* 17 237–254. 10.1111/j.1467-8551.2006.00489.x

[B75] Weiss H. Nicholas J. Daus C. (1999). An examination of the joint effects of affective experiences and job beliefs on job satisfaction and variations in affective experiences over time. *Organ. Behav. Hum. Decis. Process.* 78 1–24. 10.1006/obhd.1999.2824 10092469

[B76] Weiss H. M. Cropanzano R. (1996). “Affective events theory: a theoretical discussion of the structure, causes and consequences of affective experiences at work,” in *Research in Organizational Behavior*, eds Staw B. M. Cummings L. L. (Amsterdam: Elsevier).

[B77] Weissenfeld K. Abramova O. Krasnova H. (2019). “Antecedents for cyberloafing–a literature review,” in *Proceedings of the 14th International Conference on Wirtshaftsinformatik*, Siegen.

[B78] Wong K. F. E. Yik M. Kwong J. Y. Y. (2006). Understanding the emotional aspects of escalation of commitment: the role of negative affect. *J. Appl. Psychol.* 91 282–297. 10.1037/0021-9010.91.2.282 16551184

[B90] Wu L. -Z. Birtch T. A. Chiang F. F. T. Zhang H. (2018). Perceptions of negative workplace gossip: a self-consistency theory framework. *J. Manag.* 44, 1873–1898. 10.1177/0149206316632057

[B79] Xu E. Huang X. Jia R. Xu J. Liu W. Graham L. (2020). The “evil pleasure”: abusive supervision and third-party observers’ malicious reactions toward victims. *Organ. Sci.* 31 1115–1137. 10.1287/orsc.2019.1349 19642375

[B80] Yildiz Durak H. Saritepeci M. (2019). Occupational burnout and cyberloafing among teachers: analysis of personality traits, individual and occupational status variables as predictors. *Soc. Sci. J.* 56 69–87. 10.1016/j.soscij.2018.10.011

[B81] Yu L. Duffy M. K. (2020). The whiplash effect: the (moderating) role of attributed motives in emotional and behavioral reactions to abusive supervision. *J. Appl. Psychol.* 106 754–773. 10.1037/apl0000810 32673027

[B82] Yu L. T. Duffy M. K. (2021). The whiplash effect: the (moderating) role of attributed motives in emotional and behavioral reactions to abusive supervision. *J. Appl. Psychol.* 106 754–773. 32673027 10.1037/apl0000810

[B83] Zhang C. Yu M. C. Marin S. (2021). Exploring public sentiment on enforced remote work during COVID-19. *J. Appl. Psychol.* 106 797–810. 10.1037/apl0000933 34138587

[B84] Zhang J. Akhtar Muhammad N. Zhang Y. Sun S. (2019). Are overqualified employees bad apples? A dual-pathway model of cyberloafing. *Int. Res.* 30 289–313. 10.1108/INTR-10-2018-0469

[B85] Zhang J. L. Liu J. (2018). Is abusive supervision an absolute devil? Literature review and research agenda. *Asia Pac. J. Manag.* 35 719–744. 10.1007/s10490-017-9551-y

[B86] Zhang Y. Liu X. Chen W. (2020). Fight and flight: a contingency model of third parties’ approach-avoidance reactions to peer abusive supervision. *J. Bus. Psychol.* 35 767–782. 10.1007/s10869-019-09650-x

[B87] Zhou X. Fan L. Cheng C. Fan Y. (2020). When and why do good people not do good deeds? Third-party observers’ unfavorable reactions to negative workplace gossip. *J. Bus. Ethics* 171 599–617. 10.1007/s10551-020-04470-z

[B88] Zoghbi Manrique de Lara P. (2006). Fear in organizations. *J. Manag. Psychol.* 21 580–592. 10.1108/02683940610684418

[B89] Zoghbi-Manrique-de-Lara P. Viera-Armas M. (2017). Corporate culture as a mediator in the relationship between ethical leadership and personal internet use. *J. Leadersh. Organ. Stud.* 24 357–371. 10.1177/1548051817696877

